# 3D ITO-nanowire networks as transparent electrode for all-terrain substrate

**DOI:** 10.1038/s41598-019-41579-2

**Published:** 2019-03-21

**Authors:** Qiang Li, Zhenhuan Tian, Yuantao Zhang, Zuming Wang, Yufeng Li, Wen Ding, Tao Wang, Feng Yun

**Affiliations:** 1 Key Laboratory of Physical Electronics and Devices for Ministry of Education and Shaanxi Provincial Key Laboratory of Photonics & Information Technology, Xi’an Jiaotong University, Xi’an, Shaanxi 710049 P. R. China; 2 School of Electronic and Information Engineering, Xi’an Jiaotong University, Xi’an, Shaanxi 710049 P. R. China; 3 Department of Electronic and Electrical Engineering, University of Sheffield, Mappin Street, Sheffield, S1 3JD UK

## Abstract

A 3D ITO nanowire network with high quality by using polystyrene as an assisted material has been prepared, demonstrating superior optoelectronic performances with a sheet resistance of 193 Ω/sq at 96% transmission. Both remarkable flexibility tested under bending stress and excellent adhesion applied on special terrain substrate have been achieved. This method has led to a full coverage of micro-holes at a depth of 18 µm and a bottom spacing of only 1 µm, as well as a perfect gap-free coverage for micro-tubes and pyramid array. It has been proved that this 3D ITO nanowire network can be used as a transparent conductive layer for optoelectronic devices with any topography surface. Through the application on the micro-holes, -tubes and -pyramid array, some new characteristics of the 3D ITO nanowires in solar cells, sensors, micro-lasers and flexible LEDs have been found. Such 3D ITO nanowire networks could be fabricated directly on micro-irregular substrates, which will greatly promote the application of the heterotypic devices.

## Introduction

There is an increasing demand on micro-irregular structural components with developing intelligent systems^1–3^. It is becoming urgent to develop an effective approach to fabricate transparent conductive layer for heterotypic devices. Nowadays, graphene^4–6^, carbon nanotubes^7–9^ and silver nanowires^10–16^ have been widely used as flexible and transparent electrodes. A metal nanowire network prepared by an electrospinning method has also been applied in the fabrication of flexible touch-screen devices and transparent conducting tapes^17,18^. However, for some devices which require a special shape, such as a pyramid, rectangular groove or tubular shape, there are a great number of limitations for obtaining electrodes with a uniform and full coverage. For a device whose surface is the pyramid shape, the traditional method using a thermal evaporation or e-beam metal causes the metal on the bottom to aggregate while the metal is difficult to deposit on the top, forming a non-uniform conductive film. The same problem will be faced by spinning silver nanowires. If the graphene is used, due to the tension, it is difficult to fit closely with the surface of the substrate, forming an air gap which seriously restricts the electrical properties of the device. At the same time, for a V-shaped groove or a rectangular groove device having a width of several micro-meters and a depth of several tens of micro-meters, the above methods are difficult to achieve the coverage of the bottom and the wall, which greatly limits the electrical performance and efficiency of the device.

Indium tin oxide (ITO) has been widely used as a standard transparent electrode in various types of optoelectronic devices. Although a number of alternative material systems have been proposed in order to replace them, ITO films still remain dominant in the commercial market due to their mature manufacturing technologies^19,20^. ITO thin films are too brittle to be used in flexible devices or the devices with an irregular shape. It is expected that ITO nanowires could potentially fill the niche as result of enhanced performance and high flexibility in comparison with an ITO film. However, it is difficult to prepare a dense ITO nanowire network with good lateral conductivity by any existing growth methods, such as Au-assisted vapour-liquid-solid (VLS) growth^21,22^ and self-catalytic VLS growth^23–25^. Furthermore, the preparation process is complex and expensive, strongly restricting the applications of ITO nanowires^26^. It is therefore a great challenge to achieve a full coverage on the surface of the device with a micro-irregular shape.

Here, we report our experimental results using electron-beam(EB) to prepare a three dimensional (3D) ITO nanowire network with high quality by using polystyrene (PS) as an assisted material, demonstrating superior optoelectronic performances with a sheet resistance of 193 Ω/sq at 96% transmission. Both remarkable flexibility tested under bending stress and excellent adhesion applied on special terrain substrate have been achieved. This method has led to a full coverage of micro-holes at a depth of 18 µm and a bottom spacing of only 1 µm, as well as a perfect gap-free coverage for micro-tubes and pyramid array. It has been proved that this 3D ITO nanowire network can be used as a transparent conductive layer for optoelectronic devices with any topography surface.

## Results and Discussion

### Preparation of 3D ITO-nanowires

**Figure 1:**
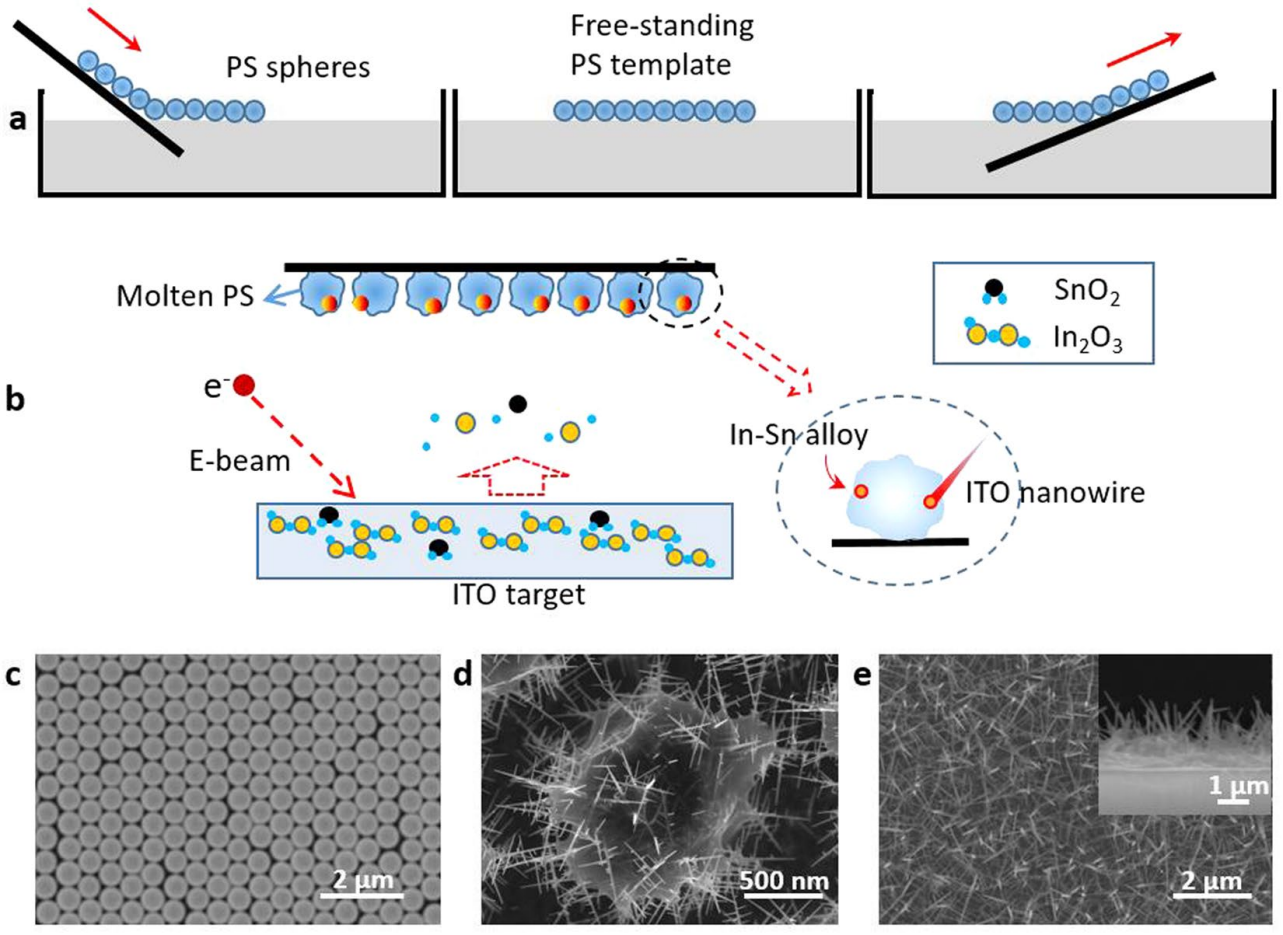
Fabrication process of ITO nanowire networks. (**a**) Self-assembly process of PS spheres on substrate. The master was treated 30 s with oxygen plasma to get a hydrophilic surface. The PS sphere dispersion slowly reached the surface of deionized water along the master. The spheres were gathered into a monolayer film by using sodium dodecyl sulfate (SDS). The substrate was inserted below the PS sphere membrane, and then was made outward by a small angle and a slow rate. (**b**) ITO source (In_2_O_3_:SnO_2_ = 90:10, wt%) was deposited on the template by e-beam at a deposition rate of 0.1 nm/s for 20 min, with the chamber temperature stabilizing at 300 °C and pressure less than 5 × 10^−4^ Pa. It took 5 min to hold the surface of PS spheres in molten state before depositing. In-Sn alloy were absorbed by the melted PS spheres, and the nanowires grew based on these nucleation point. (**c**) The PS assembly on substrate. (**d**) ITO nanowires were growing based on melted PS sphere. (**e**) The ITO nanowire network was fabricated by annealing at 470 °C for 5 min.

The process for the fabrication of a 3D ITO nanowire network is based on PS spheres. First of all, a dispersing solution with PS spheres was slowly injected the surface of the water along a hydrophilic silicon substrate, forming a monolayer which was then transferred to the surface of another required substrate and finally dry in a natural manner (Fig. 1a). Subsequently, ITO nanowires were prepared by using an electron-beam evaporation (EBE) system via PS sites in a chamber at a temperature of 280–300 °C, and the PS spheres were in a molten state. As the surface adsorption energy was high, the ITO molecules and the In-Sn alloy were adsorbed preferentially by the melted PS spheres. Nucleation can occur at the droplet/PS interface based on the In-Sn alloy, resulting in the growth of ITO nanowires (Fig. 1b). According to previous studies on the mechanism of ITO nanowires prepared by using PS as catalyst, the morphology of ITO nanowires could be controlled by tuning the diameter of PS spheres^27^. The larger the diameter of the PS sphere is, the smaller the diameter of the prepared nanowire becomes. In order to obtain the needle-shaped ITO nanowires, a single layer of PS spheres with a diameter of 670 nm was used (Fig. 1c). Because PS becomes transparent after melting, the growth state of nanowires was observed during the growth process. In Fig. 1d, after depositing 5 min, 3D ITO nanowires had been obtained on PS spheres and have begun to interweave into a network. Further increasing growth time to 25 min and then annealing at a high temperature of 470 °C, a dense 3D ITO nanowire network was formed (Fig. 1e).

**Figure 2:**
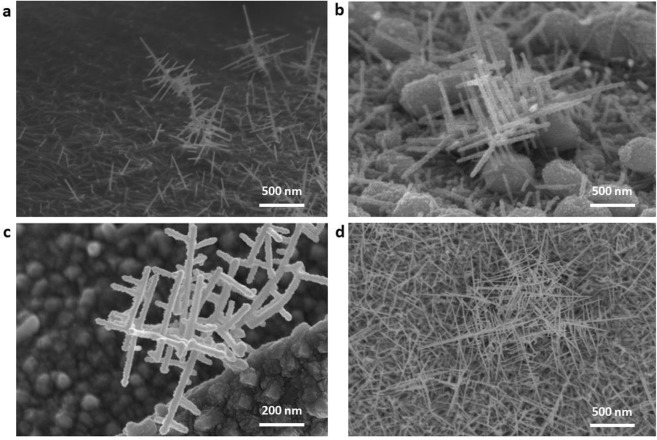
The growth morphologies of 3D ITO nanowires. (**a**) The ITO nano-trees grow on Si substrate. (**b**) The oriented growth of nanowires. (**c**) The ITO nanowires grow on Ni substrate. (**d**) The 3D ITO nanowire network.

The growth of nanowires was originally based on a trunk, then the branches were grown vertically on the trunk, and the finer nanowires could be grown on the basis of the branches again, just like a tree, showing a 3D structure (Fig. 2). Figure 2a is the 3D ITO nanowires grown on Si substrate, which shows that the branches grow vertically to the trunk, and the length of branches can exceed that of the trunk. In order to observe the oriented growth of the 3D ITO nanowires, a silver (Ag) film of 40 nm was used to cover most of surface area of PS spheres. The 3D structure was formed by the vertical growth between nanowires (Fig. 2b). Meanwhile, the 3D nanowires can also grow directly on metal substrates. Figure 2c shows the morphology of ITO nanowires grown on nickel (Ni) substrate. It can be seen that the appearance of nanowires is like a tree, with branches growing continuously. Figure 2d is the ITO nanowire network, but the 3D structure can still be seen in the original position of PS sphere. The PS material is decomposed into benzene, styrene, and other volatile gases when heated. The residual PS could be removed by annealing, and then ITO nanowires with a uniform distribution and better crystal quality can be obtained.

### Application of 3D ITO-nanowires

Generally speaking, any other methods such as the transfer method (for graphene), the spin-coating method (for Ag nanowires), or the electric-spinning method (for metal nanowires) require multiple steps in order to be completed on devices and cannot be combined well with some irregular substrates (deep V-pits, pyramid, tubular devices, etc). These drawbacks have limited the optoelectronic performance of the devices. The 3D ITO nanowire network has its own unique advantages of preparing on all terrain substrates directly and covering completely.

**Figure 3:**
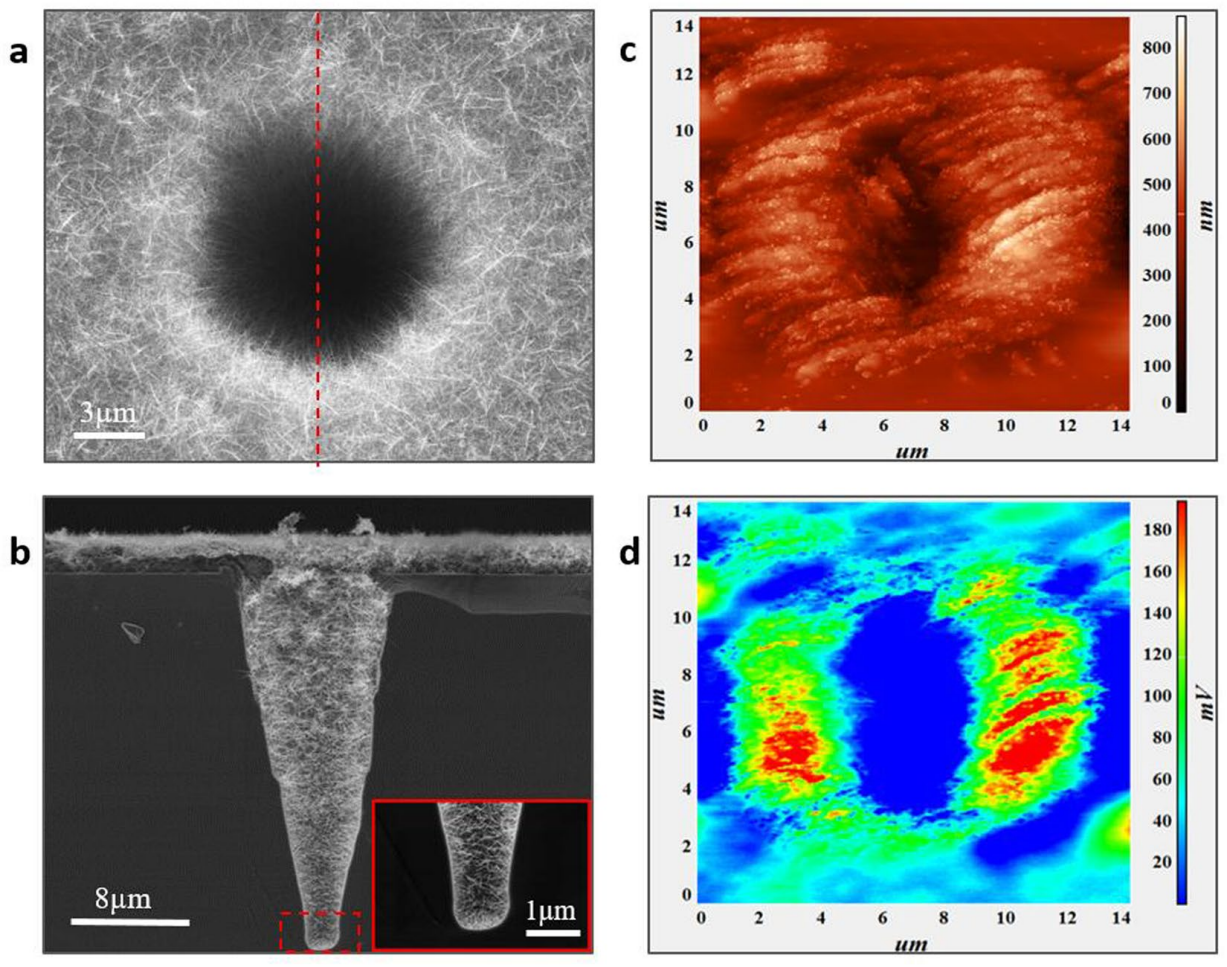
Morphology and electric field distribution of deep V-pits covered by ITO nanowire networks. (**a**) Single hole was covered by ITO nanowire networks completely. (**b**) All the inner-wall of the hole was covered by ITO nanowire networks, and the insert is the large image of bottom area. (**c**) The AFM image (measured by Contact Topography Mode) and (**d**) the electric field distribution of V-pits covered by nanowires were measured by SNOM through applying 10 *V* to the probe tip.

Firstly, 3D ITO nanowire networks applied to deep micro V-shaped micro-holes. The Q-switched nanosecond laser (λ = 355 nm, pulse duration: 40 ns, pulse repetition rate: 1 KHz, power: 0.08 W) was used to etch the sample. And then, the PS spheres were coated on the micro-hole arrayed silicon surface by self-assembly. Lastly, the ITO nanowire networks were fabricated by electron beam evaporation for 2000s. The micro-holes and the whole surfaces were covered by ITO nanowire networks (Fig. 3a). Due to the fluidity of the PS in a molten state, it’s possible to carry the grown nanowires to cover the entire micro-hole wall. In order to investigate whether the ITO nanowires can extend to the bottom, the hole should be cut-through. Figure 3b shows a cross-section image of the hole with ITO nanowire networks, confirming that the hole has been covered by nanowires completely. The ITO nanowires could be grown at the bottom of the V-pit with the spacing of only 1 μm (insert image in Fig. 3b). To the best of our knowledge, this is only the method available so far that a conductive film can be laid completely under such a deep and small spacing. This network both has good photo-permeability and electrical conductivity. The transmittance of ITO nanowire networks is above 80% in the visible band, according to the sample which were prepared by the same process on a glass substrate (Supplementary Fig. S1). The transmittance of ITO-nanowire film is lower than that of ITO film, which indicated that ITO nanowires cause light scattering. The sheet resistance is ~150 Ω/sq measured using four-probe method on the surface of micro-hole array Si.

The 3D ITO nanowires have obvious tip-discharge effect because of their needle shape. When ITO nanowires were prepared in deep V-pit, all the tip of nanowires pointed to the centre of V-pit (Fig. 3a). The scanning near-field optical microscope (SNOM, NTMDT-Ntegra Spectra SNOM) was used to test the surface electric field. Figure 3c was the morphology of a V-pit with nanowires that could be detected, and Fig. 3d presented the distribution of surface electric field. There is obvious electric field enhancement on the edge of V-pit. Because the scan depth limit is 8 μm, the probe hangs in the air when scanning the middle area of the V-pit, and the bottom of the V-pit cannot be detected. The distribution of electric field along the V-pit cannot be completely measured. But Fig. 3d had been able to explain the existence of electric field enhancement at the tip of nanowires. This V-pit with nanowires can be further explored its field emission effect, and hopefully be prepared more sensitive detectors.

**Figure 4:**
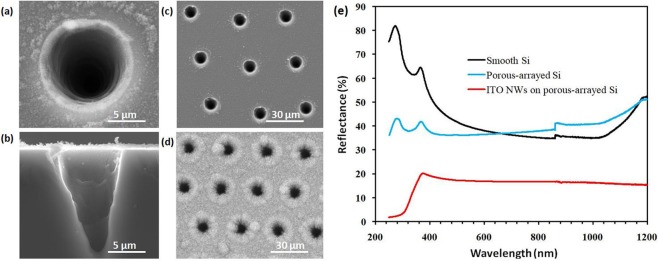
The SEM images and reflectance of μ-hole array. (**a**) A single micro-hole with the caliber of 10 μm. (**b**) The cross-sectional SEM image of a micro-hole. (**c**) The patterned area on silicon. (**d**) The μ-hole array was covered by ITO nanowire networks. (**e**) The reflectance of Si with different surfaces.

The ITO nanowire networks covering the micro-hole array could be used an excellent super-broadband antireflection layer. The plant foliage has optimized structure to absorb the solar energy after millions of years of natural evolution^28^. Based on studying the surface morphology of leaves^29^, a bionic structure was proposed. The deep micro-holes with the caliber of 10 μm and a depth of ~18 μm were ablated on silicon substrate (Fig. 4a,b) and the hole spacing is 30 μm (Fig. 4c**)**. The ITO nanowires were prepared on a micro-hole arrayed substrate with certain spacing (Fig. 4d). In this structure, the micro-hole array that was used to control the distribution of light intensity with different wavelengths was prepared by a short-pulse laser patterning process on a Si surface. ITO nanowires were then fabricated on the micro-hole arrayed surface, which could effectively reduce the scatting and the reflection of light. The reflectance reached about 15% in a band ranging from 400 to 2500 nm (Fig. 4e), and the surface could maintain a strong electrical conductivity. This method will provide a novel and practical model for obtaining a textured surface to improve the efficiency of solar cells by using the infrared band light. It could be applied to other material systems, such as InGaN or Cu based-solar cell, which will improve the utilization of infrared light effectively.

**Figure 5:**
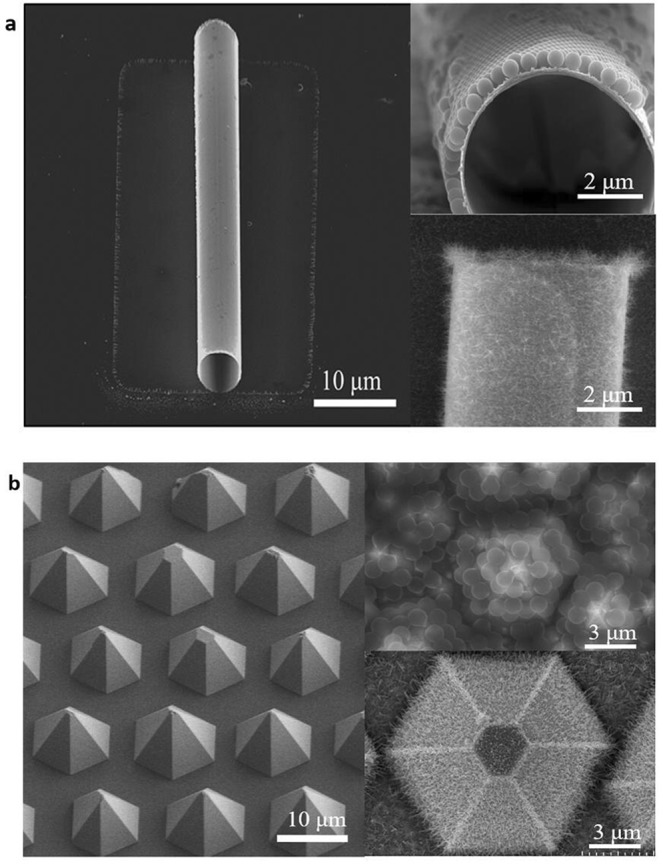
ITO nanowire networks as transparent conductive layer for micro-tube and pyramid. (**a**) The PS spheres can be spread well along the wall of GaN/InGaN micro-tube, and the ITO nanowire networks were fabricated. All the outer wall of the tube was wrapped by nanowires. (**b**) The micro-pyramid array can be covered by ITO nanowire networks without any gap.

At present, most of the spectra obtained for micro- and nano-lasers come from the photoluminescence, and there are many problems to be solved for electroluminescence^30,31^. One of the most important problems is the preparation of the electrodes. Finding a way to fabricate the electrodes and to make them fit the micro/nano devices effectively and completely without reducing the light output is currently being sought by researchers. In our previous work^32^, we have successfully fabricated micro-tubes by strain-induced self-rolling of a InGaN/GaN quantum wells nanomembrane. Freestanding quantum wells micro-tubes, with a diameter of 6 μm and wall thickness of 50 nm (Fig. 5a), were formed when the coherently strained InGaN/GaN quantum wells hetero-structure was selectively released from the hosting substrate. Periodic oscillations due to whispering-gallery modes resonance were found superimposed on photoluminescence spectra even at low optical excitation power. It has always been difficult for such devices to produce electrodes on its outer walls. We have achieved the full coating on the outer wall of the micro-tube using PS spheres, and then the 3D ITO nanowires were prepared by e-beam. A dense ITO nanowire network was formed on the outer wall of the micro-tube (Fig. 5a). This is the first time that all the outer wall of a micro-tube was covered by the transparent conductive electrode and connected to the substrate completely, laying the foundation for electroluminescent devices.

For flexible devices based on PDMS (polydimethylsiloxane) or PET (polyethylene terephthalate) substrate, to prepare transparent conductive electrodes on the surface directly is still a problem. We have demonstrated a method to obtain super flexible light-emitting diodes (LEDs) based on high quality pyramid arrays on PDMS substrate^33,34^. Ag grid and Ag nanowires were employed as the electrical connection. The performance reduction results from cracks appearing at the junction of the Ag grid and Ag nanowires. Using this method, the PS spheres were coated on the surface of the micro-pyramid array, and then 3D ITO nanowire network film was fabricated on the pyramid array LED based on PDMS substrate directly by e-beam under 280 °C for 25 min (Fig. 5b). This 3D ITO nanowire network was grown directly on the surface of the pyramid array, which has the characteristics of gap free and full bonding to the substrate as the transparent conductive electrode (Supplementary Fig. S2). At the same time, because the 3D ITO nanowire network film has a gradient refractive index and surface roughening effect, the light extraction efficiency of the device could be greatly improved without deteriorating the device turn-on voltage^27^. It was not necessary to anneal when preparing 3D ITO nanowire network for the device based on flexible substrate. The transmittance is ~70% in the visible band^35^. The 3D ITO nanowire network prepared by this method provides a perfect solution for directly fabricating electrodes based on flexible substrates.

### Properties of 3D ITO-nanowires

One of the most remarkable characteristics of the 3D ITO nanowires prepared by this method is due to the needle shape. There are no spherical particles formed on the top of nanowires, which is different from all the previous methods of preparing ITO nanowires. The growth direction is opposite to that by using previous VLS approach. The balls (In-Sn alloy particles) were located at the bottom of nanowires and wrapped by PS. The needle-shaped nanowires were grown by crystallization from bottom to top. In the traditional VLS mechanism, spherical particles are typically located at the top of nanowires and thus such nanowires are separated through a crystallization process from top to bottom (Supplementary Fig. S3). Secondly, the nanowire network prepared by this method exhibit a uniform distribution. As our ITO nanowires are fabricated using PS spheres, the regular monolayer of PS spheres ensures the uniform distribution of the formed 3D ITO nanowire network. Thirdly, our nanowires demonstrate a highly controllable diameter, which is determined by the diameter of PS sphere employed. The ITO nanowires with the smaller diameter are fabricated by using the larger PS spheres. The bigger molten PS spheres have stronger adsorption capacity. Therefore, small In-Sn alloy-droplets can be wrapped within the molten PS in order to allow nanowires grow from down to top with a needle shape.

**Figure 6:**
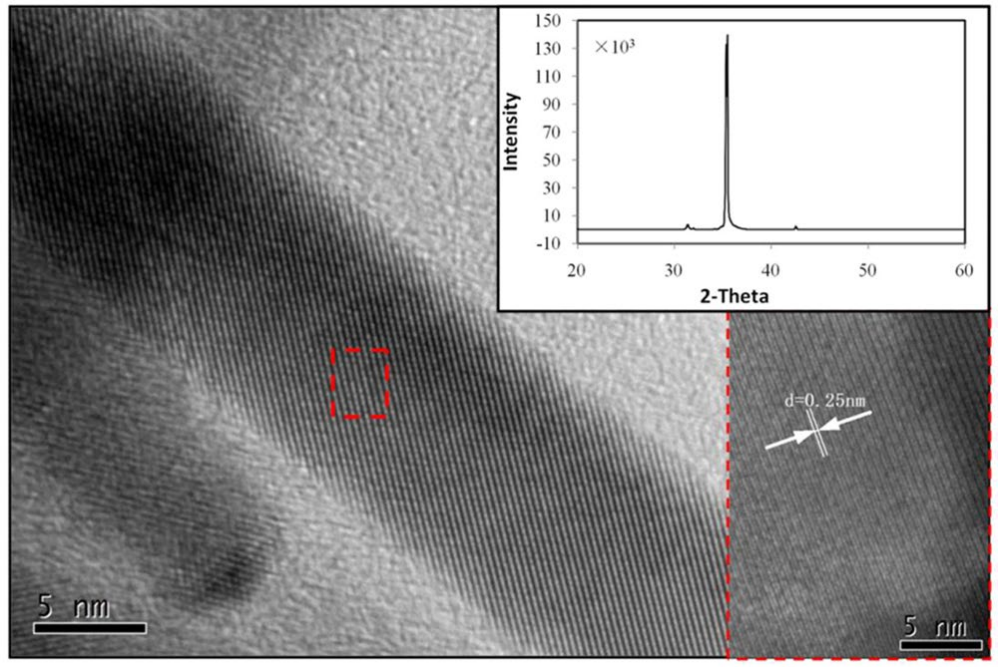
The HRTEM image of ITO nanowire. The inset is the XRD spectrum of ITO nanowire network.

Figure 6 is the high-resolution transmission electron microscope (HRTEM) of a single ITO nanowire. The result clearly shows the high degree of crystallinity with clear lattice fringes. Spacing between the lattice fringes was found to be 0.25 nm, which is well coincided with the “d” spacing of the (400) plane of the cubic phase of In_2_O_3_. The insert figure shows the XRD line profile for the ITO nanowire network. The intensity of the two major (222) and (400) peaks at 2θ = 30.5° and 35.4° in ITO NWs film is obvious. The (400)/(222) ratio in ITO nanowires is more than 100 times higher than that in ITO film, which indicates the predominant growth direction along the [100] direction^36,37^.

**Figure 7:**
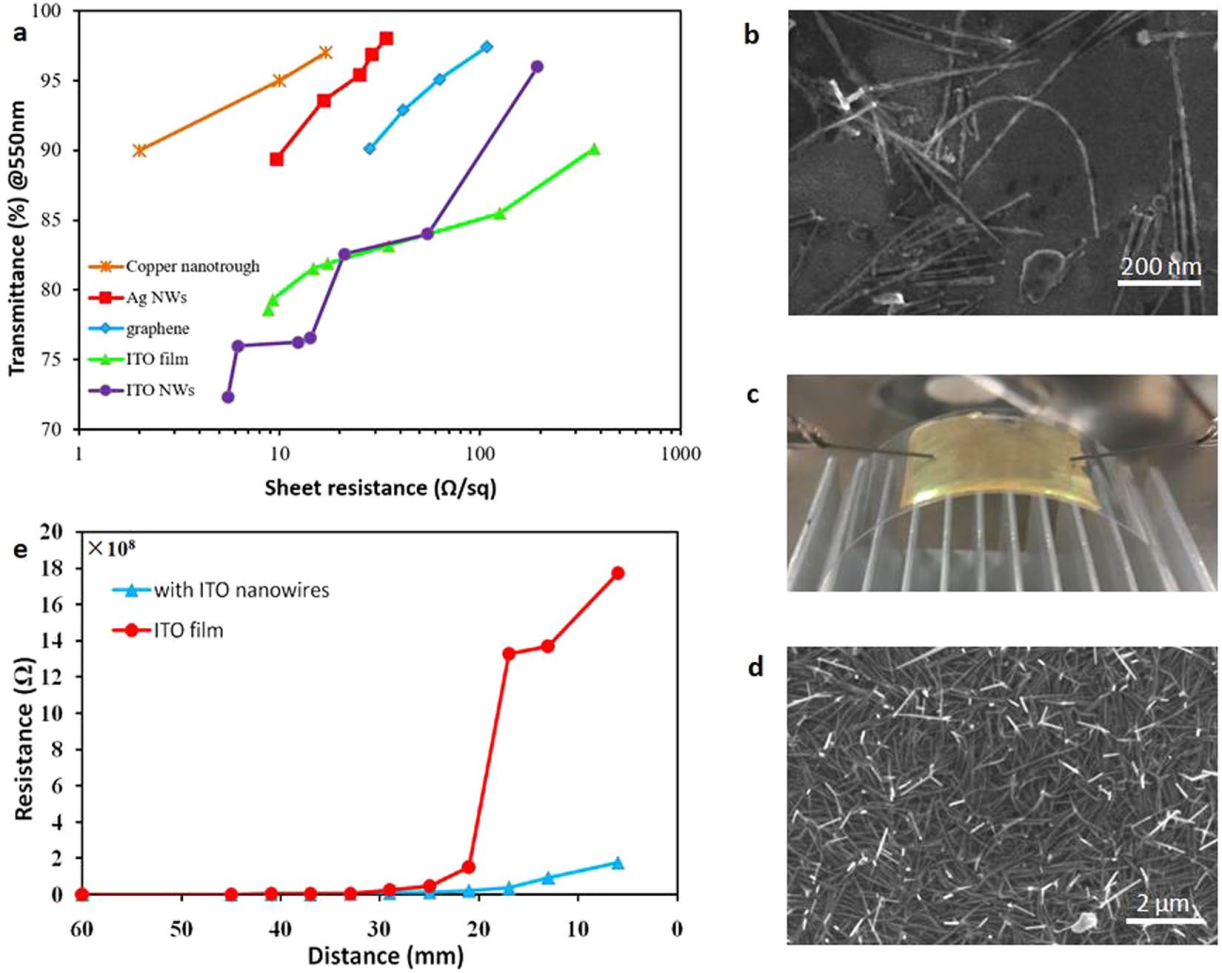
ITO nanowire networks as transparent and flexible electrode. (**a**) Sheet resistance versus optical transmission (@550 nm) for coppernanotrough^19^, silver nanowires (Ag NWs)^17^, grapheme^7^, ITO film and ITO nanowire networks. (**b**) The ITO nanowires have strong flexibility. Samples with ITO nanowires were placed in the acetone solution, shaken by sonication for 0.5 h, and then precipitated. A suspension containing ITO nanowires was obtained by removing the supernatant diluted solution. The ITO nanowire suspension was coated on silicon. (**c**) An electrical conductivity test under bending. (**d**) SEM images of the 3D ITO nanowires after being squeezed by a PET template. (**e**) Resistance versus bending radius for bendable transparent electrodes consisting of ITO nanowire networks or ITO films on 200-μm-thick PET substrates.

When the thickness of the ITO nanowire network prepared by this method is ~150 nm, the sheet resistance is 193 Ω/sq with a transmission of 96%. With increasing thickness, the density of the nanowire network increases, and the scattering of the nanowires is enhanced. When the thickness increases to ~300 nm, the sheet resistance is 5 Ω/sq and T = 73%. The performance is a little worse than other transparent conducting electrodes such as those based on copper nanotrough, graphene, and Ag nanowires, but which is close to or better than ITO film (Fig. 7a).

The 3D ITO nanowires have the abilities of bending and compression. The sample with ITO nanowires was soaked in chloroform for 30 min under ultrasonic condition. When the PS was dissolved away, ITO nanowires fell in the solution. The ITO nanowires suspension was obtained, and then a small amount of which was coated on a silicon substrate. Due to the external pressure during the coating process, a small portion of the nanowires were bent (Fig. 7b), which indicated that the ITO nanowire have strong toughness.

A layer of ITO film with a thickness of 150 nm was prepared on the PDMS substrate, and then the 3D ITO nanowire networks were fabricated on the ITO film. This sample was attached to a PET substrate with a length of 60 mm (Fig. 7c). Compared with the sample without ITO nanowire networks under bending test, the result was shown in Fig. 7e. On examining the transparent electrode after both bending regimes, we could find no obvious degradation in electrical conductivity. In contrast, severe degradation in electrical conductivity is observed in ITO films after bending to <20 mm.

In order to verify the anti-extrusion ability of nanowires, the above sample with nanowires could be re-stamped with a layer of PET on the side of the 3D ITO nanowires and was pressed by external force. In Fig. 7d, it can be seen that the entire nanowires have been bent and did not cause large-area fractures, which indicated that the nanowire networks have strong compressive resistance.

## Conclusions

In summary, we have shown that the 3D ITO nanowire networks exhibit superior optoelectronic performances, remarkable flexibility under bending stress and perfect adhesion ability for all terrain substrate. One of the most remarkable characteristics of the 3D ITO nanowires prepared by this method is due to the needle shape. There are no spherical particles formed on the top of nanowires, which is different from all the previous methods of preparing ITO nanowires. Secondly, the nanowire network prepared by this method exhibit a uniform distribution. Thirdly, our nanowires demonstrate a highly controllable diameter, which is determined by the diameter of PS sphere employed. This method has led to a full coverage of micro-holes at a depth of 18 µm and a bottom spacing of only 1 µm. This is only the method available so far that a conductive film can be laid completely under such a deep and small spacing.

The ITO nanowire networks covering the micro-hole array could be used an excellent super-broadband antireflection layer, which will provide a novel and practical model for obtaining a textured surface to improve the efficiency of solar cells by using the infrared band light. A dense ITO nanowire network was formed on the outer wall of the micro-tube (diameter of 6 μm and wall thickness of 50 nm). This is the first time that all the outer wall of a micro-tube was covered by the transparent conductive electrode and connected to the substrate completely, laying the foundation for electroluminescent devices.

Through the application on the micro-holes, -tubes and -pyramid array, some new characteristics of the 3D ITO nanowires in solar cells, sensors, micro-lasers and flexible LEDs have been found. Such 3D ITO nanowire networks could be fabricated directly on micro-irregular substrates, which will greatly promote the application of the heterotypic devices.

## Methods

### Preparation of ITO nanowire suspension

The substrate with PS spheres (670 nm) was used to grow ITO NWs by using the growth conditions for deposition 20 min. Then, this sample was soaked in chloroform for 30 min under ultrasonic condition. When the PS was dissolved away, some NWs fell in the solution without any damage. The solution was allowed to stand for an additional hour and the above solution was removed with a syringe to obtain an ITO nanowire suspension.

### Fabrication of deep V-shaped micro-holes

The silicon surface can be etched by an ultrahigh energy laser pulse in very short time. The cone-hole array was formed on the silicon surface after the laser scanning by repeating the ablation process. The spacing of micro-holes was controlled by the moving speed of platform and laser pulse repetition rate. The Q-switched nanosecond laser (λ = 355 nm, pulse duration: 40 ns, pulse repetition rate: 1 KHz, power: 0.08 W) was used to etch the sample in a fast scanning mode.

### Optical and electrical characterization

The sheet resistances of the ITO nanowire networks were measured using a digital multimeter (LDX-M-3) with a four-point probe. Bright-field transmission electron microscopy (TEM) analyses were conducted on a JEOL JEM 2100 F microscope (JEOL, Tokyo -Japan), operated at 200 kV acceleration voltage. The scanning electron microscopy (SEM) analyses were conducted on a HITACHI SU6600 Schottky Emission VPFE-SEM instrument. The X-ray diffraction (XRD) analyses were conducted on X’Pert PRO (Almelo-Netherlands). The X-ray Photoelectron Spectroscopy (XPS) analyses were carried out with a Kratos Axis Ultra DLD spectrometer using a monochromatic Al Kα source operated at 150 W. Spectra have been charge corrected to the main line of the C 1 s spectrum set to 284.3 eV. The surface electric field was tested by the scanning near-field optical microscope (SNOM, NTMDT-Ntegra Spectra SNOM) with Kelvin probe (30 nm) under AFM mode. The AFM scanning mode is Contact Topography Mode and the scanning Rate is 8000 Hz. The scanning area is 15 μm × 15 μm.

## Supplementary information

Supplementary materials
