# Supplementary material for: 3D ITO-nanowire networks as transparent electrode for all-terrain substrate
Source: Sci Rep. 2019 Mar 21;9:4983. doi: 10.1038/s41598-019-41579-2 (PMC6428818; doi:10.1038/s41598-019-41579-2)
Supplement: Supplementary file 1 — Supplementary materials [file 41598_2019_41579_MOESM1_ESM.pdf]

3D ITO-nanowire networks as transparent electrode for all-terrain substrate

Qiang Li[1,2,3], Zhenhuan Tian[2],Yuantao Zhang[2], Zuming Wang[2], Yufeng Li[2], Wen Ding[2], Tao Wang[3], and Feng Yun[1,2]*

[1]*Key Laboratory of Physical Electronics and Devices for Ministry of Education and Shaanxi Provincial Key Laboratory of Photonics & Information Technology, Xi'an Jiaotong University, Xi'an, Shaanxi, 710049, P. R. China*

[2]*School of Electronic and Information Engineering, Xi'an Jiaotong University, Xi'an, Shaanxi, 710049, P. R. China*

[3]*Department of Electronic and Electrical Engineering, University of Sheffield, Mappin Street, Sheffield, S1 3JD, UK*

Supplementary 1

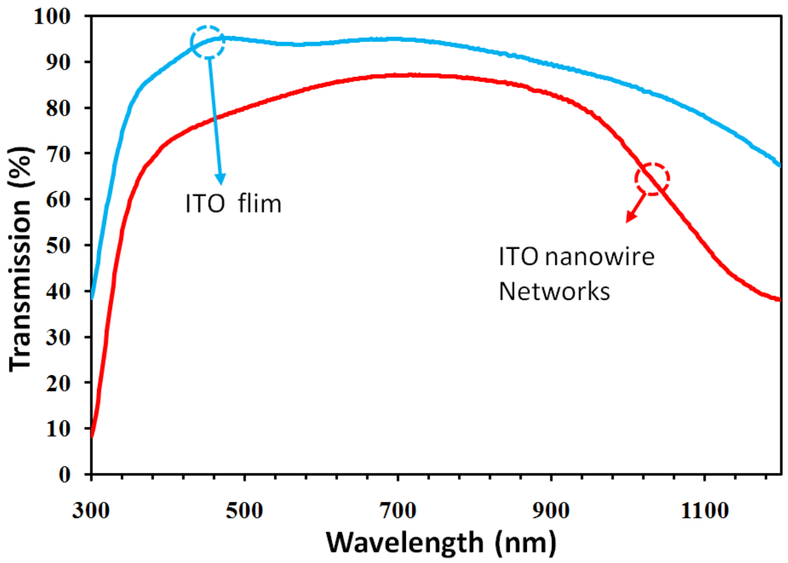

Figure S1|The transmittance of ITO-nanowire networks in the wavelength of 300-1200 nm.

Supplementary 2

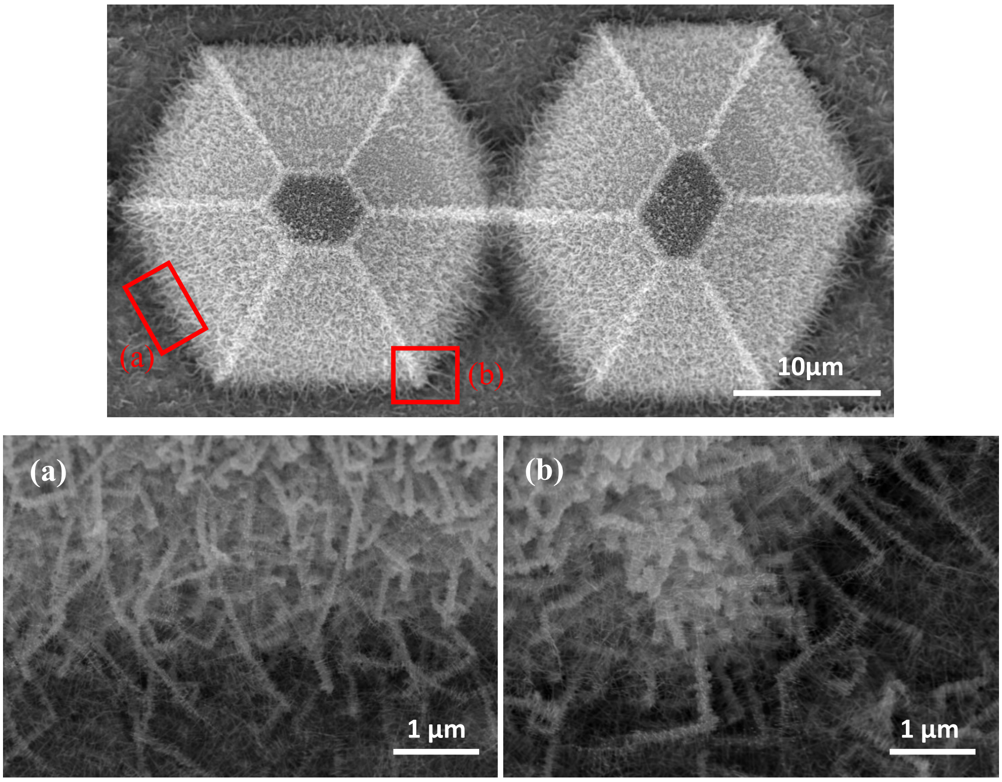

Figure S2|The 3D ITO nanowire network was grown on the surface of the pyramid array. a, The connection between the edge of pyramid and the substrate. **b,** The connection between the corner of pyramid and the substrate.

Supplementary 3

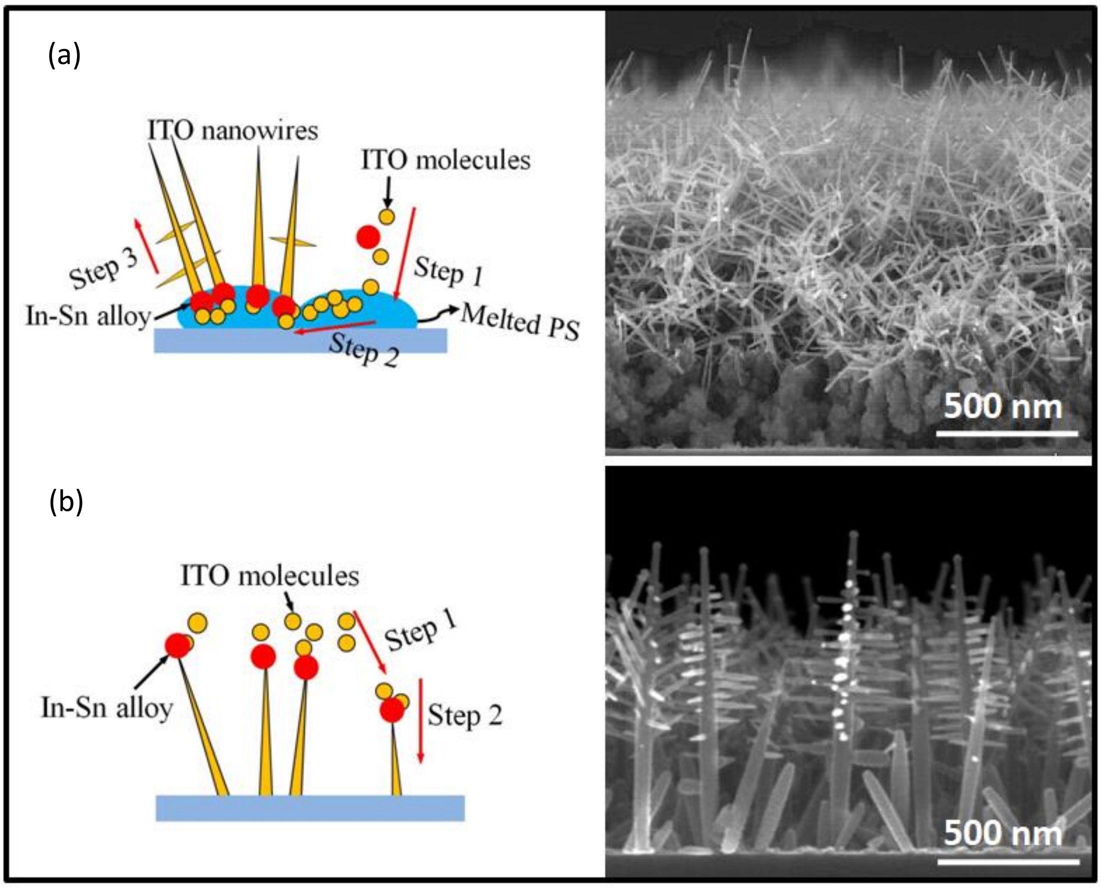

Figure S3|The growth schematic diagrams and SEM images of ITO nanowires via PS spheres and physical vapour deposition. **a,** In our method via PS,step1: ITO molecules were adsorbed by the molten PS; step 2: some molecules were adsorbed directly on the surface of In-Sn alloy; step 3: the ITO nanowires began to grow from bottom to up. From the SEM image, the ITO nanowires are interwoven into a dense network and there are no spherical particles at the top of the nanowires. **b**, In the method of physical vapour deposition, step1: ITO molecules were adsorbed directly by In-Sn alloy; step 2:the ITO nanowires began to grow from up to bottom. From the SEM image (Nanotechnology 23, 105608, 2012), the top of the nanowires has obvious spherical particles.
